# Sociodemographic Variation in Consumption Patterns of Sustainable and Nutritious Seafood in Australia

**DOI:** 10.3389/fnut.2018.00118

**Published:** 2018-12-03

**Authors:** Anna K. Farmery, Gilly A. Hendrie, Gabrielle O'Kane, Alexandra McManus, Bridget S. Green

**Affiliations:** ^1^Australian National Centre for Ocean Resources and Security, University of Wollongong, Wollongong, NSW, Australia; ^2^Commonwealth Scientific and Industrial Research Organisation, Health and Biosecurity, Adelaide, SA, Australia; ^3^Health Research Institute, University of Canberra, Canberra, ACT, Australia; ^4^Faculty of Health Sciences, Curtin University, Perth, WA, Australia; ^5^Institute for Marine and Antarctic Studies, University of Tasmania, Taroona, TAS, Australia

**Keywords:** dietary guidelines, omega-3 fatty acids, sustainable seafood, wild capture, aquaculture, nutrition

## Abstract

National dietary guidelines (DGs) consistently recommend consuming seafood for health benefits, however, the sustainability of increasing seafood consumption is often challenged. Seafood products vary in environmental performance as well as health benefits, yet there is no information integrating the health and ecological impacts of different seafood choices. The first step in optimising improved health and environmental outcomes is to examine more closely the types of seafood being consumed at population and individual levels, to develop the means to increase the intake of seafood that is optimal for human health and the environment. The purpose of this analysis was to better understand the specific types and amounts of seafood consumed by the Australian population, and by socioeconomic subgroups within the population, to determine the relative nutritional content and sustainability of seafood consumed by these groups. Secondary analysis of the Australian Health Survey (AHS) (2011–2013), which reached 32,000 people (25,000 households) was undertaken. The majority of respondents (83%) did not consume any seafood on the day of the survey. Results indicated the proportion of seafood consumers was lowest among adults who were unemployed, had the least education and were the most socio-economically disadvantaged. Crustaceans and farmed fish with low omega 3-content, such as basa and tilapia, were identified as the least nutritious and least sustainable seafood categories. These two categories constituted a substantial amount of total seafood intake for the lowest socio-economic consumers, and over 50% for unemployed consumers. In contrast, consumers in the highest socio-demographic group consumed mainly high trophic level fish (moderate nutrition and sustainability) and farmed fish with high omega-3 content (high nutrition, moderate sustainability). Fewer than 1% of adults or children reported eating seafood identified as both more nutritious and less resource intensive, such as small pelagics or molluscs. Opportunities exist to increase seafood intakes to improve health outcomes by varying current seafood consumption patterns to maximise nutritional outcomes and minimise environmental impacts. Initiatives to promote the health and environmental benefits of seafood should be promoted at the population level, with targeted interventions for specific groups, and should encourage consumption of highly nutritious low resource intensive types of seafood.

## Introduction

National dietary guidelines (DGs) consistently recommend consuming seafood (including saltwater and freshwater fish, molluscs, cephalopods, and crustaceans) for health benefits. Global seafood consumption has doubled over the past 50 years, from an average of 9.9 kg per capita in the 1960s to over 20 kg in 2016 (1). This increase has been enabled through improved availability, resulting from increased production, reduced waste, improved distribution channels, rising incomes and urbanisation (2). The increase in seafood consumption is not evenly distributed within populations or between countries, and despite the growth in consumption, DGs in many countries promote increasing seafood intake where population level consumption remains less than the amount recommended to achieve health outcomes (3, 4). In Australia, for example, the DGs promote 140–280 g of seafood per person per week, which corresponds with an increase in consumption of more than 40% to meet recommended food group intakes (3). Demand for seafood is expected to continue to increase (5) and research is therefore needed to determine how best to meet demand in a way that improves population health with the least environmental impact.

Concern over perceived negative ecological consequences of implementing recommendations to increase seafood consumption for health benefits have been raised. For example, consuming seafood has been presented as a conflict between health and environmental sustainability (6–12), while dietary recommendations to eat more fish have been criticised as potentially unsustainable (7, 13–19). However, there has been no thorough analysis of the environmental, social, and economic impacts of these recommendations. Concerns around increasing seafood consumption have focussed predominantly on overfishing of wild stocks for direct consumption or for aquaculture feed. While this issue is of critical importance, the focus on overfishing has meant that complex relationships between the environmental impacts of different fishing methods, or aquaculture practices, and the nutritional content of products has been overlooked, particularly in the broader context of sustainable diets (20).

The environmental impact of seafood varies between species and production methods (21, 22). Seafood in general has a lower environmental impact than most animal-derived farmed proteins (23–25), when measured across both ecological and abiotic variables, such as carbon footprints. Information on the environmental performance of wild fisheries and aquaculture is available through a range of public and private organisations, however, few of these also include information on human health.

Seafood products differ in terms of their nutritional content, indicating a broader discussion of seafood sustainability that includes public health and nutrition is needed (26). The health benefits linked to seafood consumption include brain function and visual development, as well as protection from chronic conditions, such as cardiovascular diseases (27–29), which are the leading causes of death globally (30). Seafood can provide a range of nutrients, including protein and long-chain omega-3 polyunsaturated fatty acids (n-3 PUFAs), and essential micronutrients (31). Many small fish species that are consumed whole with bones, heads, and viscera, such as sardines have an especially high nutritional content (32).

Awareness of the nutritional content of different seafood species is an important consideration for population health. In the US, for example, data from the National Health and Nutrition Examination Survey (NHANES) 2003–2008 food consumption survey showed that only 20% of seafood consumed was in the high omega-3 group (4) and the intake of these PUFAs was about 40% of the suggested level (33). To date, research exploring the links between nutritional value and environmental sustainability of the types of seafood consumed has not been undertaken. To our knowledge, this study is a first attempt to fill this gap by integrating the health and ecological impacts of different seafood choices.

Examining the types of seafood consumed within populations provides an opportunity to create well informed initiatives, aimed at increasing the intake of seafood using approaches that are optimal for human health and the environment. In this study, a secondary analysis of data from the Australian Bureau of Statistics (ABS) Australian Health Survey (2011–2013) was undertaken to provide an indication of the specific types of seafood Australians are consuming, and to describe the sociodemographic variation in consumption patterns. The analysis included types of seafood eaten, by age and gender, the demographic groups at risk of consuming seafood with lower nutritional profiles, and those consuming both more- or less-sustainable and nutritious seafood. Consumption trends and targeted options for improving health and sustainability outcomes, based on the findings of this research and published literature, are presented in the discussion. The primary purpose of this analysis was to develop a clearer picture of the specific types, and amounts, of seafood consumed by different sociodemographic groups in order to inform initiatives, including population level nutrition interventions, about increasing the intake of seafood that is optimal for human health and the environment.

## Methods

The most recent Australian Health Survey (AHS) (2011–2013) was conducted by the ABS in 2011–2013, reaching 32,000 people (25,000 households). The publicly reported data on fish and seafood consumed were presented in very broad categories, such as “finfish,” “crustacea and molluscs,” or “packed fish and seafood” (34). More detailed information on the specific types of fish and seafood consumed was collected as part of the survey, although these data have not, as yet, been analysed.

The secondary analysis utilised data collected from the 12,153 respondents completing the National Nutrition and Physical Activity Survey (NNPAS). Given the complexity of the AHS, a more detailed description of the sampling framework and data collection methods is available in the comprehensive “Users Guide” on the ABS website (35). Briefly, the method used to collect the dietary intake data as part of the NNPAS was two 5-phase 24-h recalls, where respondents were asked to recall the previous 24 h intake of food, beverages, and dietary supplements. The sample was randomly selected across the year, with the exception of August and September, to attempt to take account of seasonal effects on nutritional intake. Efforts to achieve a good distribution across days of the week were not highly effective, and Friday and Saturday are underrepresented in the data. For children aged < 15 years, the interview was conducted primarily with a parent or guardian, and children were encouraged to participate. Parental consent was granted to interview respondents aged 15–17 years, while some parents opted to provide this information on the child's behalf.

To improve accuracy and quality of the data collected, interviewers used an Automated Multiple-Pass Method (AMPA) developed by the United States Department of Agriculture and adapted by the ABS together with Food Standards Australia and New Zealand (FSANZ) to better reflect the Australian food supply. This method attempts to maximise respondents' memory recall and was used in conjunction with a Food Model Booklet to assist respondents in the estimation of portion size and quantities of recalled items.

Nutrient intake data were provided for each food, beverage or supplement item recalled using the AUSNUT 2011–13 food nutrient database (36). Recalled items were also coded into a hierarchy of food classification–at the major (2 digit), sub-major (3-digit), minor (5-digit) and descriptive 8 digit level. Using the hierarchy of food classification all foods that were classified as fish, or seafood, or mixed dishes where fish or seafood were the main ingredient, were identified for inclusion in this research.

### Seafood classification

Types of seafood reported by respondents were firstly classified into categories. Finfish were allocated into the categories of aquaculture and wild fishery. Crustaceans and molluscs were given their own categories, while other fish products and mixed dishes were grouped into a separate category. The wild fishery and aquaculture categories were further classified into subcategories. Wild capture finfish were divided into four categories based on commonalties between species in terms of consumer demand and ecological traits (Table 1).

**Table 1:** Justification for grouping of aquaculture and wild fisheries into subcategories.

**Category**	**Subcategory**	**Description**
Aquaculture finfish	High omega-3	Current evidence suggest that 250 mg of omega-3 LCPUFAs per week is associated with reduced cardiac mortality from coronary heart disease and reduced risk of sudden death from cardiovascular disease (3). The omega-3 content of seafood varies between species. Fish that contained 250 mg or more omega-3 per 100 g edible portion was considered to have a higher level of omega-3. Omega-3 content was sought through the AUSNUT 2011–2013 Food Nutrient Database (36).
	Low omega-3	Fish that contained < 250 mg/100 g were considered to have a lower level. Omega-3 content was sought through the AUSNUT 2011–2013 Food Nutrient Database (36).
Wild fishery finfish	High trophic	The trophic level of finfish was determined using an online database fishbase.org. Many species of popular table fish are higher order predators with trophic levels of three or more. Large, high-trophic-level predators, including tuna, shark, and swordfish, were given their own category as many of these stocks are overfished and wild capture fisheries preferentially target large, high-trophic-level species (37).
	Popular table fish	Widely consumed fish that were not categorised as large predators, small pelagic or underutilised were included as popular table fish. These species are considered more “popular” than some other types of fish due to taste, texture, smell, and colour, and, therefore, regularly purchased and consumed (38).
	Small pelagic	Pelagic fish inhabit the pelagic zone of the ocean, or the open water that is not associated with the ocean floor or the shore. Small pelagic fish include forage fish such as anchovies, sardines, and larger fish such as mackerels. Predators of small pelagics include larger fish such as tuna and billfish. Small pelagics are often highly nutritious (2).
	Underutilised species	Includes those species which are not highly targeted and are fished below the maximum sustainable yield.

Omega-3 content was used to categorise aquaculture into two groups, as it was a measure that could be determined for all the species groups recorded, regardless of the variations in production. The authors note that this grouping resulted in variation within the high omega-3 category. The nutritional content of seafood within each category was assumed to be similar for the purposes of this initial grouping.

#### Wild capture or aquaculture

Survey respondents provided common names of seafood, and these were matched to species names, where possible, based on the most commonly consumed species, noting that common names can in reality represent a number of different species. Identifying the species of animal in a meal that is processed and ready to be consumed can be difficult for consumers. This limitation is relevant to all food groups included in 24-h recall survey data, however, for seafood it can complicate results as the common names reported by the survey can in reality represent very different species.

Seafood was categorised as coming from wild capture fisheries or aquaculture, however, some seafood is sourced through both aquaculture and wild capture. In these cases, we classified such seafood based on the volume available for consumption. For example, barramundi was categorised as aquaculture given the volume of farmed barramundi available in Australia, from domestic and imported sources, is greater than wild-caught (39). Determining the source of crustaceans and molluscs, in terms of wild capture or aquaculture, was not possible as no information was provided at the species level.

#### Mixed dishes

Seafood is often consumed as part of a meal containing other foods. Many respondents reported that they consumed other fish products and mixed dishes, such as tuna mornay or stir fry with prawns, on the day of the survey. The amount of mixed dishes consumed includes the weight of all ingredients, not just the seafood as is the case for other categories.

### Data analysis

#### Secondary analysis of the australian health survey data

For the secondary analysis of the Australian Health Survey data the face to face recall (day one) was utilised. Using day 1 of the survey's dietary data meant inclusion of the entire sample of 12,153 respondents. In comparison, day 2 was completed by 64% of respondents (*N* = 7,735), therefore reducing the sample size. In addition, significant differences in energy intake reported between day 1 and 2 of the survey suggested day 2 data may be subject to mis- or under-reporting. Day one of dietary recall data were collected during face-to-face interviews and on day 2 by telephone. This differing methodology can influence the data that is collected. The first day of recall is referred to from hereon in as the day of the survey.

All data were weighted to reflect the demographic structure of the Australian population, and weighted means presented for the population and demographic descriptive statistics. Consumers of seafood were identified as those individuals who reported consuming any seafood on the day of the survey. Mean consumption among consumers only are also reported. Differences between sociodemographic subgroups were assessed for significance using Independent samples *t*-test or One-Way Analysis or Variance for differences in mean consumption in grams, or Chi-Squared for percentages of the population consuming; with significance set at a level of *p* < 0.05.

### Linking health and sustainability

Consumption data by sociodemographic group were analysed, based on the categories of seafood consumed, to provide a broad picture of the variation between groups. A more detailed analysis was then conducted to examine the sustainability as well as nutritional content of the seafood categories, beyond omega-3 content. Seafood is a source of lean protein, as well as essential nutrients. The content of protein, omega-3, calcium, zinc, iodine, and selenium per 100 g was calculated for each species (36). These nutrients were selected to use in this context as they are significant for public health in Australia and seafood has been identified as an important source (3). As these values varied in order of magnitude, the percentage contribution to estimated average requirement (EAR) or adequate intake (AI) was calculated for each nutrient category for all species (40). Percentage values were averaged to determine a “nutrition score” for each species and an average nutrition score determined for each seafood category (See Table S1). Heavy metal content and cholesterol were not included in the analysis, although these issues are presented in the discussion.

For sustainability, each seafood was scored against three categories to develop a semi-quantitative “sustainability score” for the seafood and for each category (see Table S2). The authors note that seafood sustainability, and sustainability in general, is a broad area and subject to individual values and interpretations. The categories selected, and the scoring approach, were adapted from those defined by the Marine Stewardship Council (41) and the Aquaculture Stewardship Council (42). The categories were also selected based on seafood sustainability issues identified by health practitioners (43), consumers (44), and community surveys (45). For wild capture species, the categories were stock status, resource use and ecosystem impacts. For aquaculture the categories were resource use, ecosystem impacts and health and disease management. Scoring was qualitative and based on the likelihood of the seafood achieving a high (positive) outcome for each category. A high degree of certainty equated to the species being assessed as more sustainable (score = 3), and a low degree to less sustainable (score = 1). Peer reviewed literature was used where possible to inform the assessment, in particular for imported products, as well as published reports and websites of seafood sustainability organisations. The carbon footprint was used as a proxy for resource use, as fuel use during fishing is the main source of energy use and carbon emissions in fisheries (25). The carbon footprint is used here as an indicator of energy use in aquaculture, as feed-related energy inputs typically account for a large proportion of total energy use (46). The use of this measure allows for the same assessment to be made across both wild capture and farmed seafood. The use of antimicrobials was applied as a measure of health and disease management in aquaculture. Where possible assessments of ecosystem impacts, including habitat damage, were used to inform the assessment. Where these were not available, reference to impacts or management of impacts were used to guide assessment.

## Results

### Australian population consumption of seafood

Seventeen per cent of respondents consumed seafood on the day of the survey (Table 2). When considered at an Australian population level, the average amount of seafood consumed was 26.7 g per person per day, where adults consumed 30.5 g and children 12.3 g (significance of difference *p* < 0.001). The proportion of adults consuming seafood on the day of the survey was also significantly higher than children (19.2% of adults and 11.5% of children, *p* < 0.001). Across the population as a whole, the average consumption for males was significantly higher than females (28.7 and 24.7 g, respectively, *p* = 0.003), while approximately the same numbers of males and females reported consuming seafood (16.9% and 18% of the Australian population, respectively, NS).

**Table 2:** Mean consumption (in grams) and the percentage of the sample consuming seafood by demographic characteristics for Australians adults and children (2011–2012).

		**Adults**					**Children**					**Whole population**				
		**Population**		**Consumers**			**Population**		**Consumers**			**Population**		**Consumers**		
		**Mean**	***SD***	**% popn**	**Mean**	***SD***	**Mean**	**SD**	**% popn**	**Mean**	***SD***	**Mean**	***SD***	**% popn**	**Mean**	***SD***
Gender	Male	33.2	92.6	18.5	180.3	146.1	12.4	50.2	11.9	131.7	113.7	28.7	85.7	16.9	172.0	142.3
	Female	27.9	81.0	19.8	142.3	120.8	12.2	41.4	11.0	107.3	76.5	24.7	75.0	18.0	137.8	116.7
	Total	30.5	86.9	19.2	159.1	133.9	12.3	46.1	11.5	120.1	98.4	26.7	80.5	17.5	153.4	130.0
Age group	0–17	–	–	0.0	–	–	12.3	46.1	11.5	120.1	98.4	12.3	46.1	11.5	120.1	98.4
	18–30	27.5	86.0	15.7	170.7	142.7	–	–	–	–	–	27.5	86.0	15.7	170.7	142.7
	31–50	30.3	87.2	18.3	165.3	140.5	–	–	–	–	–	30.3	87.2	18.3	165.3	140.5
	51–70	34.0	91.7	21.4	157.0	132.3	–	–	–	–	–	34.0	91.7	21.4	157.0	132.3
	71+	28.6	73.7	21.4	138.1	108.5	–	–	–	–	–	28.6	73.7	21.4	138.1	108.5
Highest level of school education	Finished high school	31.4	88.2	20.3	153.4	128.6	–	–	–	–	–	31.35	88.02	20.3	153.3	128.4
	Year 11 or below	29.3	85.4	18.1	166.0	139.8	–	–	–	–	–	27.62	82.64	17.2	163.8	137.9
Highest level of education	Postgraduate degree/Graduate diploma/Graduate certificate/Bachelor degree	31.2	84.6	21.8	145.1	124.8	–	–	–	–	–	31.24	84.65	21.8	145.1	124.8
	Advanced diploma/Diploma/Certificate I/II/III/IV	30.0	87.3	18.8	165.6	137.7	–	–	–	–	–	29.79	86.92	18.6	165.8	137.4
	No non-school qualification	30.2	88.1	17.7	163.2	136.2	–	–	–	–	–	28.34	84.91	16.8	160.2	133.9
	Level not reported	34.4	87.1	22.2	182.4	144.9	–	–	–	–	–	34.13	86.74	21.9	182.4	144.9
Employment status	Employed	31.0	89.2	19.4	163.7	136.9	–	–	–	–	–	30.32	88.13	18.9	162.9	136.2
	Unemployed	26.5	89.0	13.1	199.7	173.2	–	–	–	–	–	23.67	84.16	11.7	192.4	167.3
	Not in labour force/Not applicable	29.7	81.6	19.3	148.6	125.2	–	–	–	–	–	22.26	69.15	16.2	139.7	118.6
Household type	Without children	31.7	86.7	20.3	159.2	130.7	–	–	–	–	–	31.15	85.64	19.9	158.6	130.0
	With children	29.0	87.3	17.4	159.0	140.1	–	–	–	–	–	23.17	76.02	15.0	146.4	129.7
SEIFA–Index of Relative Socio-Economic Disadvantage (IRSD, 2011)	Lowest 20%	29.2	84.3	17.0	167.6	136.7	–	–	–	–	–	26.36	79.66	15.8	165.6	137.3
	Second quintile	28.4	82.6	18.5	164.9	146.0	–	–	–	–	–	24.83	76.70	16.8	158.7	140.6
	Third quintile	26.4	77.5	18.6	165.5	129.0	–	–	–	–	–	23.08	71.81	16.7	159.2	125.0
	Fourth quintile	31.8	91.2	18.6	149.9	127.1	–	–	–	–	–	27.62	83.34	16.6	145.5	122.2
	Highest 20%	36.0	96.7	22.6	150.9	130.0	–	–	–	–	–	31.14	88.90	20.7	143.1	125.1

#### Sociodemographic traits and proportion of population consuming seafood

A greater proportion of adults who had finished high school ate seafood than those who had not finished high school (20.3 vs. 18.1%, *p* = 0.006) or had a post graduate degree compared to the lowest level of education (21.8 vs. 17.7%, *p* = 0.001; Table 2). The proportion of the population consuming seafood on the day of the survey was highest among those with postgraduate qualifications (21.8%) and older adults (51–70 year olds 21.4% and 71+ year olds 21.4%). In contrast, the proportion of unemployed Australians consuming seafood was < 12% and the proportion of Australians under 18 years was also < 12% (Table 2). A greater proportion of adults in households without children consumed seafood than with children (20.3 vs. 17.4%, *p* < 0.001).

The Index of Relative Socio-economic Disadvantage (IRSD) is one of four measures of socio-economic indexes for areas (SEIFA), which summarises information on the economic and social conditions of people and households within an area (47). The IRSD index includes measures of relative disadvantage only, and the highest quintile reflects the areas with a relative lack of disadvantage in general. Almost 21% of individuals or households in the highest IRSD quintile consumed seafood while < 16% of individuals or households in the lowest IRSD quintile consumed seafood on the day of the survey (difference in percentage by IRSD quintiles was significant, *p* < 0.001; Table 2).

#### Sociodemographic traits and quantity of seafood consumed by population

The amount of seafood consumed per capita per day did not differ based on education or employment level. The amount of seafood consumed on the day of the survey was not different among adults who had finished high school (31.4 g) compared to those who had not (29.3 g), or between those with a postgraduate degree (31.2 g) than those with a diploma (30 g) or lower qualification (30.2 g). The volume of seafood consumed by employed and unemployed adults did not differ (31 g c.f. 26.5 g, NS).

### Consumption trends among seafood consumers

The above section summarised the per capita consumption of seafood. Here we focus on the trends within those respondents who did consume seafood (17% of total respondents). Among these consumers of seafood, the average amount consumed was 153.4 g of seafood on the day of the survey (Table 2). The amount adults reported consuming (170.7 g) was higher than reported for children (120.1 g, *p* < 0.001). The amount of seafood consumed decreased with age in adults, with the 71 + years category reporting the lowest consumption for adults (138.1 g).

Even though a lower proportion of adults who had not finished high school ate seafood, their average portion consumed was higher than adults who had finished school (163.8 g c.f. 153.3 g, *p* = 0.046). The same trend was evident in the IRSD category where the lowest quintile accounted for the smallest proportion of seafood consumers, yet individual consumers in this quintile reported eating larger amounts of seafood on average than those in higher quintiles. There was no difference in the portion size of unemployed and employed adults (199.7 g c.f. 163.7 g, NS; Table 2).

### Types of seafood consumed

#### Trends in seafood consumed in australia

**Figure 1:**
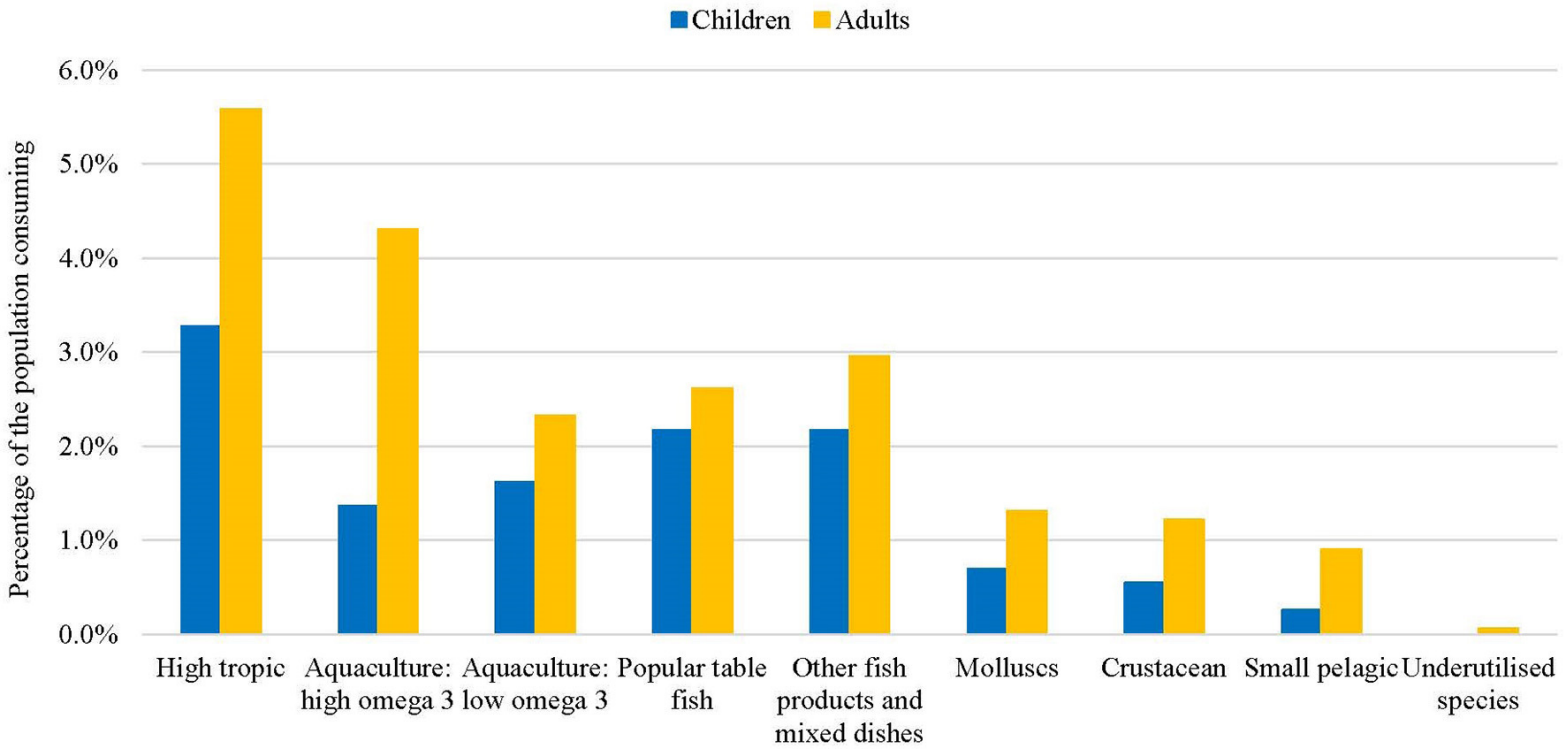
Percentage of the population consuming different fish species, for adults and children (2011–2012).

At a population level, the most commonly consumed seafood was large high trophic wild-capture fish (5.6% adults and 3.3% children) (Figure 1). For adults, the second most frequently consumed seafood was high omega-3 aquaculture fish (4.3%) and popular table fish, such as flathead and snapper, and other fish products or mixed dishes for children (2.2 and 2.2%). Fewer than 1% of all adults and children reported eating small pelagics, such as sardines and mackerel, or underutilised species such as milkfish and mullet. Around 1% of adults reported consuming molluscs or crustaceans, and fewer than 1% of children. Higher omega-3 aquaculture raised fish were consumed by 4.3% of adults and lower omega-3 aquaculture raised fish by 2.3%. Children consumed similar percentages of low and high omega-3 aquaculture fish (1.6 and 1.4%; Figure 1).

#### Trends in seafood consumed by fish eaters

**Figure 2:**
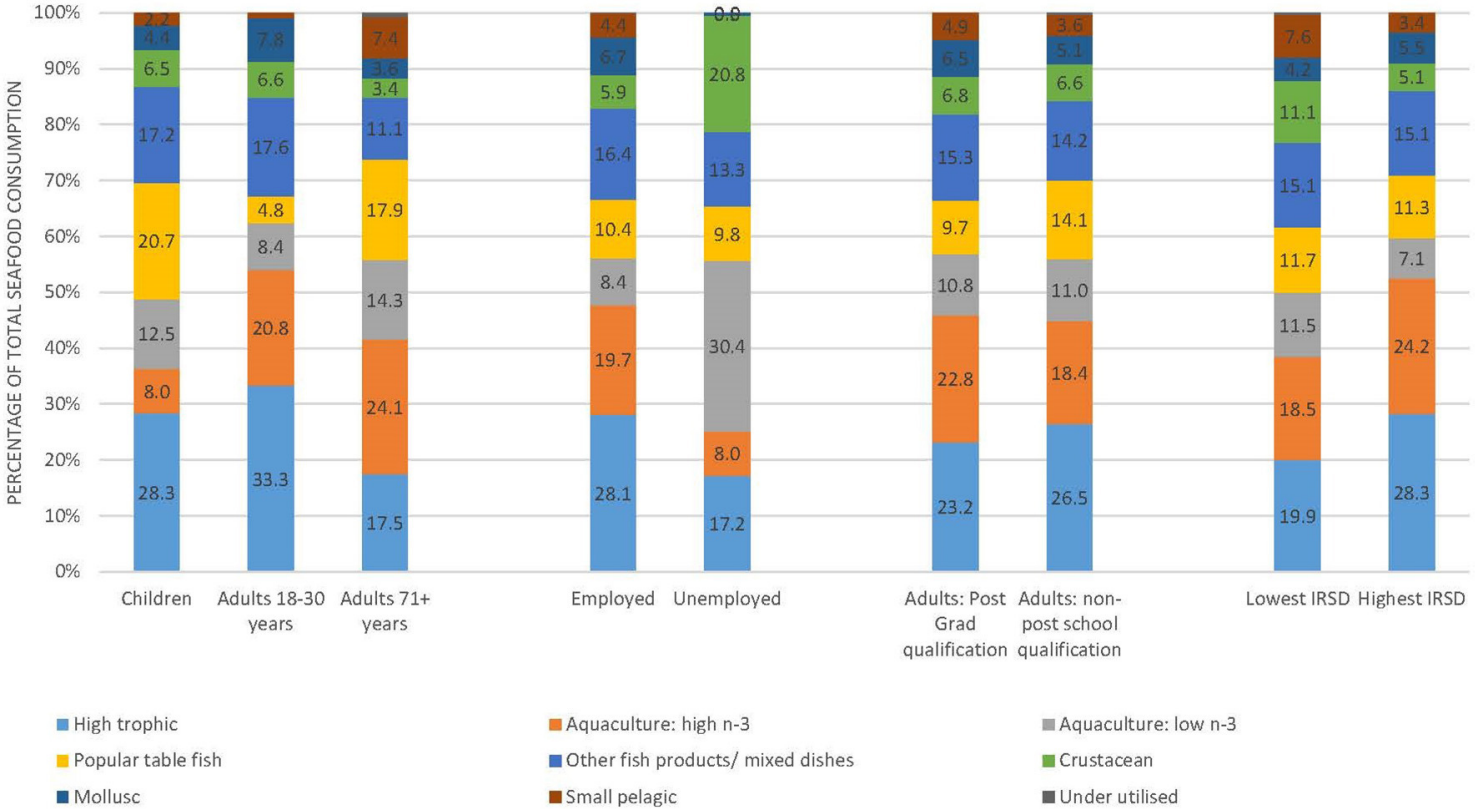
Contribution of different species groups to total seafood consumption among consumers, for key contrasting demographic categories (2011–2012).

Among adults (18–30 years) who consumed finfish, almost 21% of the total amount consumed was from the high omega-3 aquaculture group and over 33% was from the wild capture large high trophic group (Figure 2). The large high trophic fish reportedly consumed were predominately tuna (88% of high trophic, data not shown), as well as shark and swordfish. While consumption of small pelagics was low at a population level, and within the fish consumer group, small pelagics accounted for around 7.5% of seafood consumption for adults 71 + years and in the lowest IRSD quintile group.

In adults, 34% of seafood consumed was low omega-3 species such as basa and 66% was high omega-3 species, predominantly salmon (73.7%). Low omega-3 aquaculture fish accounted for over 30% of seafood consumed by unemployed respondents. This contribution was higher than for employed individuals and those not in the labour force (*p* < 0.05). Crustaceans accounted for over 20% of total seafood intake in unemployed Australians, however, due to the large variation in intake these differences were not statistically significant between employment status groups. Fish from the high omega-3 aquaculture and high trophic groups, predominantly tuna and salmon, were eaten by all consumer groups, in particular by adults 18–30 years and those in the highest SEIFA quintile, where over half of all seafood consumption was from these two groups (Figure 2). High omega-3 aquaculture accounted for a greater proportion of total consumption for female (22.8%) than male adults (17.7%, *p* < 0.01). Adult women also favoured molluscs over men (7.3%, 4.2%, *p* < 0.05), as well as more small pelagics (5.4%, 4.1%, *p* < 0.01) (data not shown).

Among children who consumed fish, on average 28.3% was high tropic fish, of which 85% was tuna, 20.7% was popular table fish, 20.5% aquaculture fish, and 17.2% was other fish and mixed dishes (Figure 2). Of the aquaculture fish consumed by children 61% was low omega-3 species, predominantly basa, and 39% was high omega-3 species, predominantly salmon (69%) and barramundi (23%). Among the consumers of “other fish products and mixed dishes,” children were more likely to choose dishes such as salmon or tuna mornay (24% total mixed dish consumption) whereas adults consumed dishes such as stir fries that contain seafood (data not shown).

Crustaceans and molluscs accounted for < 7% each of total seafood consumption in adults and children. Crustacean consumption, predominantly prawns, was highest in the low IRSD group (11.1%) and lowest in adults 71+ years (3.4%). Adults 18–30 had the highest proportion of mollusc consumption (7.8%) while for adults 71+ years molluscs accounted for a similar proportion of total seafood consumption as crustaceans (3.6%).

### Nutritional content and sustainability of seafood consumed by sociodemographic groups

**Figure 3:**
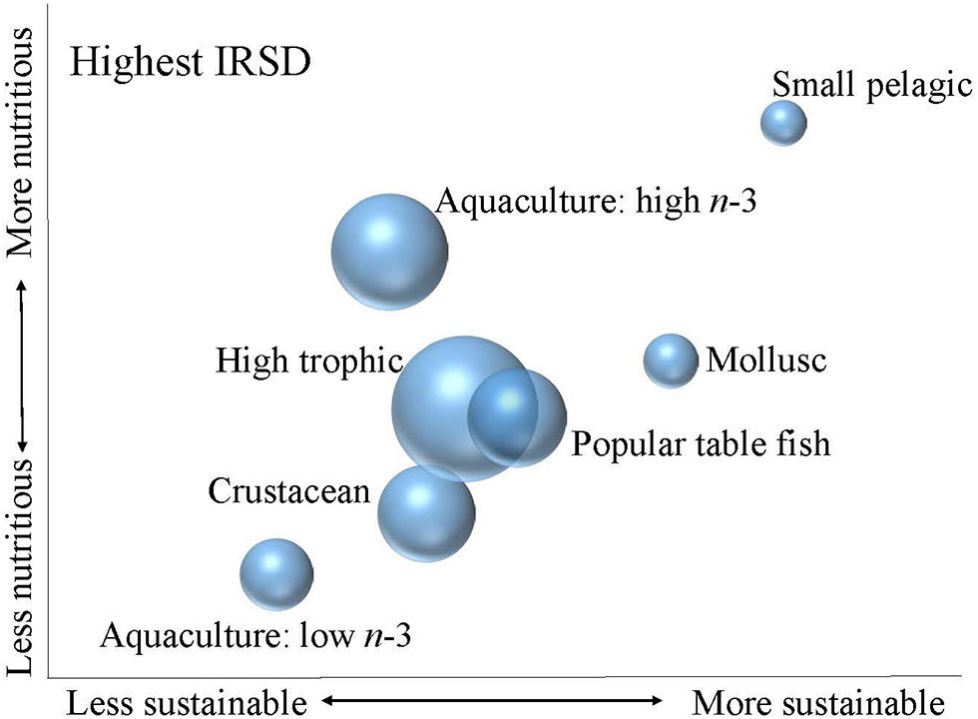
Relative nutritional quality and sustainability of seafood categories consumed by highest IRSD consumer group. The size of the bubble indicates the contribution of each seafood category to total seafood consumption within the consumer group, noting that total seafood consumption in the highest IRSD group was higher than in the lowest IRSD group.

**Figure 4:**
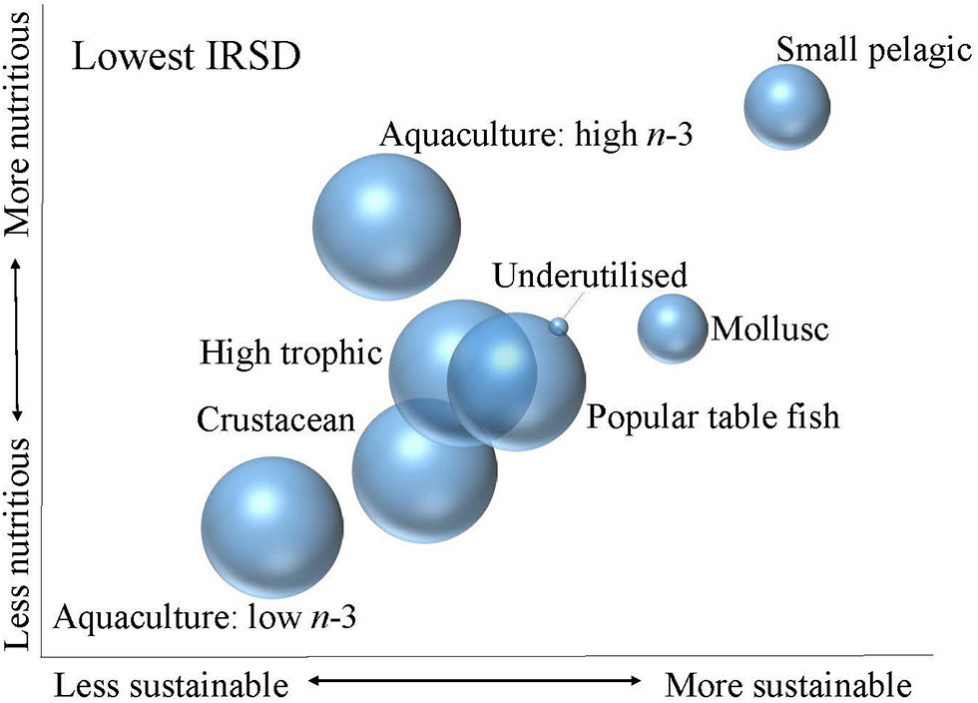
Relative nutritional quality and sustainability of seafood categories consumed by lowest IRSD group. The size of the bubble indicates the contribution of each seafood category to total seafood consumption within the consumer group, noting that total seafood consumption in the lowest IRSD group was lower than in the highest IRSD group.

More detailed analysis revealed sociodemographic variation in the consumption patterns of seafood in terms of the nutritional quality and relative sustainability of seafood (Figures 3, 4).

At a population level, the highest IRSD group consumed more seafood than the lowest IRSD group (Table 2). Within the highest IRSD group, seafood consumption was largely high trophic species, which were assessed as moderate in terms of both nutrition and sustainability, and high omega-3 aquaculture fish, which were assessed as high nutrition and moderate sustainability (Figure 3). The highest IRSD group consumed very limited amounts of the small pelagic and molluscs category. Molluscs were assessed as moderate to high for nutrition and high sustainability, while the small pelagic group was assessed high for both nutrition and sustainability. Consumption of seafood within the lowest IRSD group had a more even distribution across the categories of popular table fish, low and high omega-3 aquaculture, high tropic and crustaceans (Figure 4). Molluscs and small pelagics contributed to a small proportion of seafood consumption in this group, although small pelagics contributed to a greater share of the seafood consumed within the lowest than the highest IRSD group.

Low omega-3 aquaculture fish comprised a greater share of seafood consumption within the lowest IRSD group than highest IRSD group. This seafood category was assessed as lower both nutritionally and sustainably than all other categories. Similarly, crustaceans comprised a greater share of seafood consumption within the lowest than highest IRSD group and were also assessed as relatively low for both sustainability and nutrition. Shellfish is also moderately high in cholesterol, although high consumption has not been linked to adverse cardiovascular events (48). Underutilised species contributed marginally to overall consumption within the low IRSD group, and made negligible contribution within the highest group. The underutilised species group was assessed as moderate in terms of both sustainability and nutrition, which was similar to popular table fish and high trophic species group assessments.

## Discussion

The results of this analysis of a nationally representative sample of the Australian population showed that, on the day surveyed, most people (over 83%) did not eat any fish or seafood, and that there were distinct sociodemographic variations in consumption patterns. The lowest sociodemographic group, and in particular unemployed people, were less likely to consume seafood, and the seafood they did consume tended to be from the least nutritious and least sustainable categories. In contrast, the highest sociodemographic group, in particular those with post graduate qualifications, were more likely to consume seafood, and the types they ate were moderately to highly nutritious and moderately sustainable. Seafood consumers appear to consume amounts consistent with recommendations (3), however, future initiatives could focus on increasing consumption in those individuals who currently don't consume seafood, as well as promote the differences in nutrition and sustainability between different types of seafood.

### Consumption trends and targeted options for improving health outcomes in different demographic groups

#### Consumption trends for adults and children

The proportion of seafood consumers was lowest among individuals who were unemployed, had the least education and were the most socioeconomically disadvantaged. These groups are also more at risk from non-communicable diseases, such as heart disease (49), the incidence of which may be reduced through seafood consumption (27, 28). Our findings are consistent with research from the United States (US) and in the United Kingdom (UK) which found that the highest seafood intakes were among the highest socio-economic groups (50, 51). In the current study, consumption was particularly low among unemployed people, potentially due to the perception that seafood is expensive (38). The least sustainable and least nutritious seafood categories, crustaceans and low omega-3 aquaculture fish, contributed more to the seafood intake of those living in the most socially and economically disadvantaged areas. This was particularly evident within the unemployed group where these two categories contributed to half of total seafood consumption. These types of seafood may provide a cost effective and accessible form of protein, but may not deliver the best nutritional or environmental outcomes.

Less than twelve percent of children reportedly consumed seafood, which is consistent with international findings reporting few children meet recommended intakes (52, 53). This finding is particularly concerning as fish consumers tend to have better diet quality (53) and regular seafood consumption has been strongly linked to optimal cognitive development (54). Children's dietary patterns are influenced by parental preferences (55) and role modelling of parents may be particularly important in regard to the consumption of seafood. The presence of bones and the (real or perceived) odour of the fish have been reported as barriers to consumption (55, 56). However, to overcome any negative attitudes towards seafood consumption, it is critical to expose children early and regularly to positive seafood experiences (57). High trophic fish, predominantly tuna, and popular table fish were commonly consumed by Australian children. While tuna is nutritionally high, several organisations have recommended that children should limit tuna and other higher trophic-level predatory fish that can accumulate high levels of mercury (58–60). Children also reported eating mixed dishes and aquaculture fish species with a low nutritional profile, which are likely to be fried fish portions or crumbed fish “nuggets” high in saturated fats, if previous Australian research provides a reasonably accurate snapshot of Australian family seafood consumption habits (61).

#### Options for improving health outcomes

Improving the availability of healthier seafood options and increasing exposure of children to seafood early and regularly, while their food preferences are being established, is one way to encourage consumption. This is also important for adults, as increasing people's confidence and establishing a habit of purchasing and preparing seafood on a regular basis are important strategies to increasing consumption (38, 57, 62, 63).

Initiatives to promote seafood consumption should also consider the nutritional requirements of specific population groups and promote seafood species based on their nutritional profile, quality, acceptability, affordability, and availability (64). For example, affordability might be a barrier to consumption in the lower socioeconomic groups, therefore promoting cheaper options with high nutritional value such as small pelagics might be a mutually beneficial strategy. The findings of this research would suggest there may be less resistance to this strategy in lower socioeconomic groups as small pelagics account for a greater proportion of total consumption in this group than others. Previous experience with purchasing and preparing fish is an important component of consumers' behaviour and their intention to eat fish (65).

Another sub-group of the population that requires consideration are seniors as they are at greater nutritional risk than the general population (66). While this research demonstrated that the percentage of seniors consuming seafood was higher than other groups, the amount consumed was lower. Reasons for lower consumption may include reduced appetite and affordability of seafood; as well as other factors which place them at nutritional risk more generally, such as decreases in sensations of taste and smell and poor dental health (67). However, diet may play an important part in promoting health and delaying the time to onset and slowing the progression of disease such as Alzheimer's Disease–for which specific dietary recommendation is regular consumption of oily fish (68). Strategies around purchasing and preparing seafood are important predictors of consumers' behaviour and their intention to eat fish, and should form part of any nutrition interventions around seafood. Interestingly, it was older Australians, and men, who ate more underutilised and pelagics, which tend to be oiler species, so there may be opportunities to promote the consumption of a broader range of less popular seafood species for people in their senior years.

### Consumption trends and targeted options for improving sustainability outcomes

Most of the information on seafood sustainability published by government fishery assessment bodies and conservation groups does not consider metrics on human health and nutrition quality. Similarly, dietary advice for seafood tends to focus on the health benefits and does not consider associated sustainability considerations. As a consequence, information integrating the health and ecological impacts of different fish choices is lacking (69). This type of information is important given that integrated messages on health and environmental sustainability can trigger behavioural changes in favour of both public and environmental health (70).

#### Sustainability concerns

High trophic fish, predominantly tuna, were commonly consumed by Australians. Australia is a major market for canned tuna, along with the US, the EU, Egypt and Japan (71). The convenience of canned tuna is promoted by companies and supermarkets (71). Tuna consumption is also encouraged through dietary advice in Australia (43). Globally, 64% of tuna stocks have healthy biomass levels (72), however, illegal, unregulated, and unreported (IUU) fishing has been problematic in several tuna fisheries (73). In addition, some species of tuna commonly used in canning, such as big eye tuna, are experiencing overfishing (72) and are listed as vulnerable by the IUCN (74). Eco-labelling is helping consumers to source tuna from fisheries that are harvesting sustainably, however, many schemes focus on a single conservation issue (75), and exclude broader sustainability issues. For example, pole and line-caught tuna is promoted due to reduced amounts of bycatch, yet this method requires more fuel use than others, resulting in a higher carbon footprint than the much larger purse seine fleets (76). It is important to note that eco-certification of seafood does not replace fisheries management tasks like stock assessment and licences/quota allocation (77). Increasing population level consumption of tuna will contribute to health outcomes, however, ensuring that the product is sustainably sourced is important.

Amongst the seafood consumers in this sample, salmon was also commonly consumed. Like tuna it is a good source of omega-3 (36), however, salmon farming is reliant on feed which is resource intensive (78, 79). Farming can have a detrimental effect on the ecosystem, including impacts on the flora and fauna around the farm, and impacts on wild fish populations (80, 81). Given the high nutritional quality of salmon, only small amounts need to be eaten to meet dietary recommendations. In contrast, larger amounts of farmed fish basa (pangasius) would need to be consumed to meet dietary recommendations for seafood, as the nutritional quality of basa is lower (36). Basa also has high resource use and environmental impacts as a result of feed, water and energy use (82). Aquaculture fish have varied nutritional profiles, resulting, in part, from the type of feed used in their production. The quantity of wild fish in feed is often used as a measure of resource use and sustainability for aquaculture, however, the environmental benefits of replacing wild fish with terrestrial products has yet to be quantified (83).

Generally crustaceans were not as commonly consumed by Australians as fish, yet, for unemployed respondents crustaceans accounted for over 20% of their total seafood consumption. The main type of crustacean consumed were prawns (including shrimp), although details were not available about the species of prawn eaten, or if they were wild caught or farmed, or domestic or imported. Regardless, crustaceans are an ecologically intensive seafood. Wild capture crustacean fisheries are fuel intensive, and while they account for only 6% of global seafood landings, they produce over 22% of greenhouse gas emissions (25). Similarly, farmed shrimp can result in substantial greenhouse gas emissions (GHG) emissions (22) as well as emissions of both eutrophying and acidifying substances (84). Therefore, the health and sustainability implications of promoting increased consumption of crustaceans needs to be considered.

#### Opportunities for consuming more sustainable and nutritious seafood

Dietary change has been recommended as an important and necessary strategy to reduce the environmental impact of the food system (22, 85–87). Dietary shifts toward consumption of low impact seafood could offer substantial environmental benefits and should be considered by health professionals as well as by public policy makers (88). For seafood, low-input aquaculture and non-trawling fisheries have much lower GHG emissions than trawling fisheries (89). For example, mussels were assessed as relatively high for sustainability as well as being nutritious. Fish and other seafood sourced from low-input, multitrophic systems could also present more sustainable options (90). Small pelagics, such as anchovies and sardines, are associated with a very low carbon footprint (25) and very high nutritional profile (36). New initiatives to make these types of seafood more appealing to consumers and to improve availability are required. Approximately one third of marine fishery landings are still used for non-food purposes, predominantly small pelagics for use in aquaculture and livestock feeds (2). Increasing the human consumption of fish destined for animal feed is a clear opportunity to increase the consumption of highly nutritious and more sustainable seafood without the need to increase catches (91).

Shifting fishing effort away from highly targeted stocks through greater consumption of underutilised species has also been promoted as a way to reduce pressure on overfished species (7, 92). While only a very limited range of underutilised species were reported as consumed in this study, underutilised species include those that are less popular and unwanted by markets, and are either not fished commercially, or caught as bycatch in other targeted fisheries. For fisheries managed by quota, increased consumption of underutilised species may not directly reduce catches of the more popular species. However, greater consumption of these species, in place of environmentally intensive species, may present a more sustainable option for increasing seafood intake. The additional benefit of these species may be their cost, which will need to be promoted, as well as increasing public awareness of benefits of these species, and ways to prepare them so they taste good.

Seafood differs to other protein sources because of its favourable fatty acid profile. Given the low proportion of people in Australia, and other countries including the US, the UK, Mexico, Hungary and South Korea, meeting the dietary recommendations for omega-3 (4, 51, 53, 93), advice to increase seafood consumption to meet these recommendations should promote health benefits at the population level, but not at the expense of environmental health. However, universal approaches such as population-wide health promotion campaigns have the potential to be inequitable in their impact (94). In the case of promoting seafood consumption, inequity may lead to uptake by existing seafood consumers, rather than across the population. Targeted approaches may be needed in order to tailor the messages to different sociodemographic groups.

Despite the low proportion of seafood consumers in many countries, global demand for seafood continues to increase, in particular, demand for more ecologically intensive forms of fish, such as farmed Atlantic salmon and crustaceans. In Australia and the US, salmon, tuna and shrimp/prawns are among the top seafood consumed (33, 43), and in the US approximately half of all seafood consumption is shrimp (50). Consumer preferences for tuna and salmon, in particular, is likely to continue given their affordability and convenience (95) and the development of new products to increase market share (96). Consumers, health professionals and policy makers need to advocate for ongoing improvements in the sustainability of these species, given the strong demand and the importance of the nutritional benefits they can provide (26). Policies aimed at moderating demand for ecologically intensive products may be necessary (97) in combination with targeted approaches to improve consumption of healthy and sustainable species.

## Limitations and further research

The use of day 1 of the Australian Health Survey (AHS) (2011–2013) data meant the entire sample of respondents were included in the secondary analysis. However, using 1 day only limits consideration of intra-individual variability of weekly consumption, and results may underestimate eating occasions. The CSIRO Healthy Diet Score survey uses a different method of dietary assessment to assess usual intake of different foods, and includes questions on frequency and amount of seafood usually consumed (98). Survey data from over 145,000 Australians using this self-reported method suggests a similar result to those reported here using 1 day intake from the Australian Health Survey. Friday and Saturday are reportedly underrepresented in the data, which may also have resulted in underestimation of consumption, given that these may be times when more people consume fish. Conversely, the inability to separate seafood from mixed dishes containing seafood may overestimate fish consumption. This research is the first secondary analysis of seafood consumption in Australia and further analysis is required to determine the influence of these limitations on consumption data.

The study was limited to the types of seafood recalled by seafood consumers. A broader study of a wider range of seafood would provide more insight into the relationship between particular species and their nutritional profile or relative sustainability. The specific nutrients and minerals selected for inclusion in this type of analysis may vary depending on the context and the relevant public health needs. A more detailed analysis could also consider a comprehensive nutrient profile for the seafood examined [see for e.g., (99)]. Results indicated a link between nutrient profile and sustainability, which is consistent with research highlighting a relationship between healthy and sustainable food, meals and diets (100, 101). The inter-relationships between the environmental and health effects of food is a relatively new field of research (102). Further exploration of this relationship in this study was not possible due to the qualitative nature of the sustainability scoring. A quantitative examination of the relationship between nutrition and sustainability in a broad range of seafood will be a valuable next step to create well informed initiatives aimed at increasing the intake of seafood using approaches that are optimal for human health and the environment.

## Conclusions

Increasing seafood consumption to meet dietary recommendations is an important element of improving health outcomes, in particular for lower socioeconomic groups and for people who currently consume little or no seafood. There are opportunities to increase intakes by varying current seafood consumption patterns to maximise nutritional outcomes and minimise ecological impacts, and more research is needed in this area. Initiatives to increase awareness of the nutritional variation and trade-offs in sustainability of different seafood types are also required. These initiatives must actively promote the health and environmental benefits of seafood at the population level, and should encourage consumption of highly nutritious, low resource intensive, types of seafood.

## Author contributions

AF and GO contributed to the original project concept and design. GH performed statistical analysis. AF wrote the first draft of the manuscript. GO, GH, AM, and BG all made written and editorial contributions, including data interpretation.

### Conflict of interest statement

The authors declare that the research was conducted in the absence of any commercial or financial relationships that could be construed as a potential conflict of interest.
