# Supplementary material for: Sociodemographic Variation in Consumption Patterns of Sustainable and Nutritious Seafood in Australia
Source: Front Nutr. 2018 Dec 3;5:118. doi: 10.3389/fnut.2018.00118 (PMC6287033; doi:10.3389/fnut.2018.00118)
Supplement: Supplementary file 1 [file Table_1.DOCX]

Supplementary Material

**Sociodemographic factors influence on the consumption of sustainable and nutritious seafood – the case of Australia**

Anna K Farmery*, Gilly A Hendrie, Gabrielle O’Kane*, Alexandra McManus, Bridget S Green

*** Correspondence:** Corresponding Authors: [afarmery@uow.edu.au](mailto:afarmery@uow.edu.au), gokane@coordinare.org.au

Table S1 Percentage contribution of 100g edible portion seafood to estimated average requirement (EAR) or adequate intake (AI) of nutrients

| **Seafood category** | **Seafood category number** | **Seafood name** | **Protein** | **Omega-3** | **calcium** | **iodine** | **selenium** | **zinc** | **Nutrition score** | **Category nutrition score** |
| --- | --- | --- | --- | --- | --- | --- | --- | --- | --- | --- |
| Aquaculture high omega | 1 | Barramundi | 42.16 | 180.85 | 2.75 | 22.10 | 59.54 | 3.38 | 51.80 | 141.55 |
|  | 1 | Cod | 40.73 | 184.78 | 2.95 | 81.54 | 63.08 | 4.99 | 63.01 |  |
|  | 1 | Nile perch | 40.55 | 171.10 | 2.72 | 21.92 | 56.92 | 3.38 | 49.43 |  |
|  | 1 | Salmon | 42.87 | 1621.92 | 9.48 | 14.61 | 44.69 | 5.61 | 289.86 |  |
|  | 1 | Trout | 42.36 | 1403.15 | 2.74 | 21.07 | 43.91 | 8.62 | 253.64 |  |
| Wild fishery – high trophic | 2 | Shark | 52.08 | 25.46 | 1.17 | 11.88 | 44.07 | 3.53 | 23.03 | 87.52 |
|  | 2 | Swordfish | 44.24 | 797.55 | 0.63 | 8.53 | 109.23 | 7.42 | 161.27 |  |
|  | 2 | Tuna | 45.57 | 329.33 | 3.21 | 25.95 | 60.20 | 5.28 | 78.26 |  |
| Wild fishery – popular table fish | 3 | Blue eye trevalla | 35.88 | 210.83 | 1.17 | 29.51 | 47.49 | 3.52 | 54.73 | 84.43 |
|  | 3 | Blue grenadier (Hoki) | 31.64 | 134.09 | 1.30 | 6.33 | 97.76 | 3.13 | 45.71 |  |
|  | 3 | Bream | 42.86 | 911.42 | 2.75 | 22.68 | 85.62 | 6.82 | 178.69 |  |
|  | 3 | Flathead | 40.79 | 153.83 | 6.23 | 7.74 | 69.41 | 5.98 | 47.33 |  |
|  | 3 | Flounder | 27.64 | 267.54 | 2.57 | 18.87 | 50.05 | 3.58 | 61.71 |  |
|  | 3 | Garfish | 35.18 | 545.87 | 2.40 | 16.53 | 42.62 | 4.36 | 107.83 |  |
|  | 3 | Gemfish | 33.18 | 405.44 | 1.05 | 15.23 | 105.00 | 3.27 | 93.86 |  |
|  | 3 | Grouper | 41.73 | 226.44 | 3.38 | 30.12 | 66.12 | 5.39 | 62.19 |  |
|  | 3 | John dory | 44.41 | 155.99 | 2.45 | 18.92 | 96.38 | 4.75 | 53.82 |  |
|  | 3 | Ling | 40.95 | 122.08 | 4.18 | 4.58 | 66.15 | 8.52 | 41.08 |  |
|  | 3 | Morwong | 38.55 | 632.48 | 3.95 | 63.33 | 69.23 | 4.09 | 135.27 |  |
|  | 3 | Mulloway | 42.41 | 491.85 | 3.13 | 5.20 | 67.00 | 4.64 | 102.37 |  |
|  | 3 | Orange roughy | 32.64 | 30.05 | 1.00 | 26.03 | 112.38 | 2.27 | 34.06 |  |
|  | 3 | Silver Perch | 43.27 | 1228.95 | 0.10 | 21.05 | 59.04 | 9.57 | 227.00 |  |
|  | 3 | Snapper | 44.09 | 268.14 | 13.47 | 31.14 | 63.92 | 7.47 | 71.37 |  |
|  | 3 | Trevally | 45.45 | 306.90 | 2.18 | 17.52 | 67.42 | 7.50 | 74.50 |  |
|  | 3 | Whiting | 40.91 | 104.67 | 4.04 | 9.12 | 95.43 | 8.42 | 43.76 |  |
| Wild fishery – small pelagic | 4 | Anchovy | 46.18 | 615.52 | 16.70 | 20.00 | 104.77 | 26.36 | 138.26 | 185.83 |
|  | 4 | Herrings | 25.82 | 1188.56 | 7.70 | 18.67 | 90.77 | 4.55 | 222.68 |  |
|  | 4 | Mackerel | 39.53 | 1431.51 | 1.34 | 101.29 | 63.02 | 5.11 | 273.63 |  |
|  | 4 | Sardines | 37.24 | 1268.63 | 7.57 | 32.96 | 80.89 | 9.90 | 239.53 |  |
|  | 4 | Whitebait | 38.18 | 201.19 | 3.80 | 23.93 | 58.62 | 4.73 | 55.08 |  |
| Wild fishery – underutilised species | 5 | Milk fish | 46.45 | 463.71 | 0.10 | 18.17 | 52.46 | 8.36 | 98.21 | 104.56 |
|  | 5 | Mullet | 37.27 | 481.45 | 5.15 | 9.17 | 122.27 | 10.18 | 110.91 |  |
| Aquaculture low omega | 6 | Basa | 33.89 | 54.91 | 0.28 | 21.05 | 36.55 | 2.76 | 24.91 | 30.64 |
|  | 6 | Tilapia | 36.82 | 98.89 | 0.10 | 18.23 | 56.77 | 7.45 | 36.38 |  |
| Crustacean | 7 | Crab | 27.45 | 75.05 | 12.10 | 39.20 | 47.69 | 42.18 | 40.61 | 51.67 |
|  | 7 | Lobster | 33.91 | 98.86 | 3.85 | 41.63 | 32.77 | 25.36 | 39.40 |  |
|  | 7 | Moreton bay bugs | 89.45 | 274.40 | 5.05 | 46.07 | 43.23 | 34.77 | 82.16 |  |
|  | 7 | Prawns | 38.12 | 108.09 | 10.26 | 19.65 | 76.63 | 14.23 | 44.50 |  |
| Mollusc | 8 | Mussels | 30.77 | 531.98 | 11.43 | 131.22 | 134.42 | 23.91 | 143.95 | 103.99 |
|  | 8 | Octopus | 25.82 | 119.35 | 5.10 | 14.22 | 64.27 | 13.70 | 40.41 |  |
|  | 8 | Oyster | 25.33 | 619.01 | 10.33 | 105.33 | 96.41 | 337.27 | 198.95 |  |
|  | 8 | Scallops | 28.45 | 209.76 | 2.50 | 93.78 | 44.82 | 21.35 | 66.78 |  |
|  | 8 | Squid | 31.14 | 288.65 | 1.58 | 13.77 | 71.63 | 12.39 | 69.86 |  |

Table S2 Sustainability rating for seafood categories

| **Seafood category** | **Seafood category number** | **Seafood name** | **Stock status** | **Resource use (CO2)** | **Habitat/ ecosystem** | **Health and disease** | **Sustainability score** | **Category sustainability score** | **References** |
| --- | --- | --- | --- | --- | --- | --- | --- | --- | --- |
| Aquaculture high omega | 1 | Barramundi |  | 2 | 2 | 2 | 2.00 | 1.93 | (Hall et al., 2011; Peet, 2014; Lin et al., 2017) |
|  | 1 | Cod |  | 2 | 2 | 2 | 2.00 |  | (Cromey et al., 2009; FAO, 2018) |
|  | 1 | Nile perch |  | 3 | 1 | 3 | 2.33 |  | (Lake Victoria Fisheries Organization, 2015) |
|  | 1 | Salmon |  | 2 | 2 | 1 | 1.67 |  | (Wilson et al., 2009; White, 2012; Parker, 2017; Watts et al., 2017; White et al., 2017; Miranda et al., 2018) |
|  | 1 | Trout |  | 2 | 2 | 1 | 1.67 |  | (d'Orbcastel et al., 2009; Watts et al., 2017) |
| Wild fishery – high trophic | 2 | Shark | 2 | 2 | 2 |  | 2.00 | 2.00 | (Tuck et al., 2013; Parker et al., 2015; Davidson et al., 2016; Stewardson et al., 2016; Patterson et al., 2017; Parker et al., 2018) |
|  | 2 | Swordfish | 2 | 2 | 2 |  | 2.00 |  | (Tuck et al., 2013; Parker et al., 2015; Patterson et al., 2017; Pons et al., 2017; Parker et al., 2018) |
|  | 2 | Tuna | 2 | 2 | 2 |  | 2.00 |  | (Hall and Roman, 2013; Patterson et al., 2017; Pons et al., 2017; Parker et al., 2018) |
| Wild fishery – popular table fish | 3 | Blue grenadier | 3 | 2 | 2 |  | 2.33 | 2.24 | (Tuck et al., 2013; Parker et al., 2015; Stewardson et al., 2016; Patterson et al., 2017; Parker et al., 2018) |
|  | 3 | Blue eye trevalla | 3 | 2 | 2 |  | 2.33 |  | (Tuck et al., 2013; Parker et al., 2015; Stewardson et al., 2016; Patterson et al., 2017; Parker et al., 2018) |
|  | 3 | Bream | 3 | 2 | 3 |  | 2.67 |  | (Parker et al., 2015; Stewardson et al., 2016; Patterson et al., 2017; Parker et al., 2018) |
|  | 3 | Flathead | 3 | 2 | 2 |  | 2.33 |  | (Tuck et al., 2013; Parker et al., 2015; Stewardson et al., 2016; Patterson et al., 2017; Parker et al., 2018) |
|  | 3 | Flounder | 2 | 2 | 2 |  | 2.00 |  | (Earl and Ye, 2016; Goodfishbadfish, 2018) |
|  | 3 | Garfish | 2 | 2 | 3 |  | 2.33 |  | (Parker et al., 2015; Stewardson et al., 2016; Patterson et al., 2017; Parker et al., 2018) |
|  |  | Gemfish | 1 | 2 | 2 |  | 1.67 |  | (Parker et al., 2015; Patterson et al., 2017; Parker et al., 2018) |
|  | 3 | Grouper | 2 | 2 | 2 |  | 2.00 |  | (Edgar et al., 2014; Goodfishbadfish, 2018) |
|  | 3 | John dory | 3 | 2 | 2 |  | 2.33 |  | (Tuck et al., 2013; Parker et al., 2015; Patterson et al., 2017; Parker et al., 2018) |
|  | 3 | Ling | 3 | 2 | 2 |  | 2.33 |  | (Tuck et al., 2013; Parker et al., 2015; Stewardson et al., 2016; Patterson et al., 2017; Parker et al., 2018) |
|  | 3 | Morwong | 2 | 2 | 3 |  | 2.33 |  | (Parker et al., 2015; Stewardson et al., 2016; Patterson et al., 2017; Parker et al., 2018) |
|  | 3 | Mulloway | 3 | 2 | 3 |  | 2.67 |  | (Parker et al., 2015; Stewardson et al., 2016; Patterson et al., 2017; Parker et al., 2018) |
|  | 3 | Orange roughy | 1 | 2 | 3 |  | 2.00 |  | (Parker et al., 2015; Stewardson et al., 2016; Patterson et al., 2017; Parker et al., 2018) |
|  | 3 | Silver Perch | 1 | 2 | 2 |  | 1.67 |  | (Diggles, 2017) |
|  | 3 | Snapper | 3 | 2 | 2 |  | 2.33 |  | (Tuck et al., 2013; Parker et al., 2015; Stewardson et al., 2016; Patterson et al., 2017; Parker et al., 2018) |
|  | 3 | Trevally | 2 | 2 | 2 |  | 2.00 |  | (Tuck et al., 2013; Parker et al., 2015; Stewardson et al., 2016; Patterson et al., 2017; Parker et al., 2018) |
|  | 3 | Whiting | 3 | 2 | 3 |  | 2.67 |  | (Parker et al., 2015; Stewardson et al., 2016; Patterson et al., 2017; Parker et al., 2018) |
| Wild fishery – small pelagic | 4 | Mackerel | 3 | 2 | 3 |  | 2.67 | 2.87 | (Tuck et al., 2013; Parker et al., 2015; Stewardson et al., 2016; Patterson et al., 2017; Parker et al., 2018) |
|  | 4 | Sardine | 3 | 3 | 3 |  | 3.00 |  | (Tuck et al., 2013; Parker et al., 2015; Stewardson et al., 2016; Patterson et al., 2017; Parker et al., 2018) |
|  | 4 | Whitebait | 2 | 3 | 3 |  | 2.67 |  | (DPI NSW, 2009; Goodfishbadfish, 2018) |
|  | 4 | Anchovy | 3 | 3 | 3 |  | 3.00 |  | (FAO, 2016) |
|  | 4 | Herrings | 3 | 3 | 3 |  | 3.00 |  | (FAO, 2016) |
| Wild fishery – underutilised species | 5 | Milk fish | 3 | 2 | 2 |  | 2.33 | 2.33 | (Meynecke et al., 2008) |
|  | 5 | Mullet | 2 | 2 | 3 |  | 2.33 |  | (Stewardson et al., 2016) |
| Aquaculture low omega | 6 | Basa |  | 2 | 2 | 1 | 1.67 | 1.67 | (Pelletier and Tyedmers, 2010; Bosma et al., 2011; Budiati et al., 2013; Huysveld et al., 2013; Rico and Van den Brink, 2014; Nhu Thuy et al., 2015) |
|  | 6 | Tilapia |  | 2 | 2 | 1 | 1.67 |  | (Budiati et al., 2013; Hall et al., 2013; Rico and Van den Brink, 2014) |
| Crustacean | 7 | Crab | 2 | 1 | 3 |  | 2.00 | 2.02 | (Parker et al., 2015; Stewardson et al., 2016; Parker et al., 2018) |
|  | 7 | Lobster | 2 | 1 | 3 |  | 2.00 |  | (Parker et al., 2015; Stewardson et al., 2016; Parker et al., 2018) |
|  | 7 | Moreton bay bugs | 3 | 1 | 3 |  | 2.33 |  | (Stewardson et al., 2016; Parker et al., 2018) |
|  | 7 | Prawns/shrimp | 3 | 1 | 2 | 1 | 1.75 |  | (Cao et al., 2011; Rico et al., 2013; Tuck et al., 2013; Farmery et al., 2015; Jonell and Henriksson, 2015; Stewardson et al., 2016; Patterson et al., 2017; Parker et al., 2018; Poore and Nemecek, 2018) |
| Mollusc | 8 | Mussels |  | 3 | 3 | 3 | 3.00 | 2.60 | (Chinabut et al., 2006; Iribarren, 2010) |
|  | 8 | Octopus | 2 | 3 | 3 |  | 2.67 |  | (Vázquez-Rowe et al., 2012; Stewardson et al., 2016) |
|  |  | Oyster |  | 3 | 3 | 3 | 3.00 |  | (Alvarenga et al., 2012; Pernet et al., 2016) |
|  | 8 | Scallops | 2 | 1 | 2 |  | 1.67 |  | (Parker et al., 2015; Stewardson et al., 2016; Patterson et al., 2017; Parker et al., 2018) |
|  | 8 | Squid | 3 | 2 | 3 |  | 2.67 |  | (Parker et al., 2015; Stewardson et al., 2016; Patterson et al., 2017; Parker et al., 2018) |

**References**

Alvarenga, R.A.F.d., Galindro, B.M., Helpa, C.d.F., and Soares, S.R. (2012). The recycling of oyster shells: An environmental analysis using Life Cycle Assessment. *Journal of Environmental Management* 106**,** 102-109. doi: <https://doi.org/10.1016/j.jenvman.2012.04.017>.

Bosma, R., Anh, P., and Potting, J. (2011). Life cycle assessment of intensive striped catfish farming in the Mekong Delta for screening hotspots as input to environmental policy and research agenda. *The International Journal of Life Cycle Assessment* 16(9)**,** 903-915. doi: 10.1007/s11367-011-0324-4.

Budiati, T., Rusul, G., Wan-Abdullah, W.N., Arip, Y.M., Ahmad, R., and Thong, K.L. (2013). Prevalence, antibiotic resistance and plasmid profiling of Salmonella in catfish (Clarias gariepinus) and tilapia (Tilapia mossambica) obtained from wet markets and ponds in Malaysia. *Aquaculture* 372**,** 127-132.

Cao, L., Diana, J.S., Keoleian, G.A., and Lai, Q. (2011). Life Cycle Assessment of Chinese Shrimp Farming Systems Targeted for Export and Domestic Sales. *Environmental Science & Technology* 45(15)**,** 6531-6538. doi: 10.1021/es104058z.

Chinabut, S., Somsiri, T., Limsuwan, C., and Lewis, S. (2006). Problems associated with shellfish farming. *Rev. sci. tech. Off. int. Epiz* 25(2)**,** 627-635.

Cromey, C.J., Nickell, T.D., Treasurer, J., Black, K.D., and Inall, M. (2009). Modelling the impact of cod (Gadus morhua L.) farming in the marine environment—CODMOD. *Aquaculture* 289(1)**,** 42-53. doi: <https://doi.org/10.1016/j.aquaculture.2008.12.020>.

d'Orbcastel, E.R., Blancheton, J.-P., and Aubin, J. (2009). Towards environmentally sustainable aquaculture: Comparison between two trout farming systems using Life Cycle Assessment. *Aquacultural Engineering* 40(3)**,** 113-119.

Davidson, L.N.K., Krawchuk, M.A., and Dulvy, N.K. (2016). Why have global shark and ray landings declined: improved management or overfishing? *Fish and Fisheries* 17(2)**,** 438-458. doi: doi:10.1111/faf.12119.

Diggles, B. (2017). *Fish facts: Silver perch* [Online]. Available: <http://www.fishingworld.com.au/news/fish-facts-silver-perch> [Accessed 13 July 2018].

DPI NSW (2009). "Whitebait - Sandy Sprat (Hyperlophus vittatus)", in: *Wild Fisheries Research Program.* DPI NSW).

Earl, J., and Ye, Q. (2016). "Greenback Flounder (Rhombosolea tapirina) Stock Assessment Report 2014/15". PIRSA, SARDI).

Edgar, G.J., Stuart-Smith, R.D., Willis, T.J., Kininmonth, S., Baker, S.C., Banks, S., et al. (2014). Global conservation outcomes depend on marine protected areas with five key features. *Nature* 506**,** 216. doi: 10.1038/nature13022

<https://www.nature.com/articles/nature13022#supplementary-information>.

FAO (2016). "The state of world fisheries and aquaculture: contributing to food security and nutrition for all". (Rome: Food and Agriculture Organzation of the United Nations, Rome).

FAO (2018). *Cultured Aquatic Species Information Programme: Gadus morhua* [Online]. Available: <http://www.fao.org/fishery/culturedspecies/Gadus_morhua/en> [Accessed 13 July 2018].

Farmery, A., Gardner, C., Green, B.S., Jennings, S., and R, W. (2015). Life cycle assessment of wild capture prawns: expanding sustainability considerations in the Australian Northern Prawn Fishery. *Journal of Cleaner Production* 87**,** 96-104.

Goodfishbadfish (2018). *Seafood and sustainability* [Online]. Available: <http://goodfishbadfish.com.au> [Accessed 13 July 2018].

Hall, M., and Roman, M. (2013). Bycatch and non-tuna catch in the tropical tuna purse seine fisheries of the world. *FAO fisheries and aquaculture technical paper* (568)**,** I.

Hall, S.J., Delaporte, A., Phillips, M.J., Beveridge, M., and O’Keefe, M. (2011). "Blue Frontiers: Managing the Environmental Costs of Aquaculture. The WorldFish Center, Penang, Malaysia".).

Hall, S.J., Hilborn, R., Andrew, N.L., and Allison, E.H. (2013). Innovations in capture fisheries are an imperative for nutrition security in the developing world. *Proceedings of the National Academy of Sciences* 110(21)**,** 8393-8398.

Huysveld, S., Schaubroeck, T., De Meester, S., Sorgeloos, P., Van Langenhove, H., Van linden, V., et al. (2013). Resource use analysis of Pangasius aquaculture in the Mekong Delta in Vietnam using Exergetic Life Cycle Assessment. *Journal of Cleaner Production* 51(0)**,** 225-233. doi: <http://dx.doi.org/10.1016/j.jclepro.2013.01.024>.

Iribarren, D. (2010). Life Cycle Assessment of fresh and canned mussel processing and consumption in Galicia (NW Spain) *Resources, conservation and recycling* 55(2)**,** 106.

Jonell, M., and Henriksson, P.J.G. (2015). Mangrove–shrimp farms in Vietnam—Comparing organic and conventional systems using life cycle assessment. *Aquaculture* (0). doi: <http://dx.doi.org/10.1016/j.aquaculture.2014.11.001>.

Lake Victoria Fisheries Organization (2015). "Nile Perch Fishery Management Plan for Lake Victoria 2015 - 2019", in: *SF/ 2015/ 49.* Indian Ocean Commission).

Lin, H.-L., Shiu, Y.-L., Chiu, C.-S., Huang, S.-L., and Liu, C.-H. (2017). Screening probiotic candidates for a mixture of probiotics to enhance the growth performance, immunity, and disease resistance of Asian seabass, Lates calcarifer (Bloch), against Aeromonas hydrophila. *Fish & Shellfish Immunology* 60**,** 474-482. doi: <https://doi.org/10.1016/j.fsi.2016.11.026>.

Meynecke, J.O., Lee, S.Y., and Duke, N.C. (2008). Linking spatial metrics and fish catch reveals the importance of coastal wetland connectivity to inshore fisheries in Queensland, Australia. *Biological Conservation* 141(4)**,** 981-996. doi: <https://doi.org/10.1016/j.biocon.2008.01.018>.

Miranda, C.D., Godoy, F.A., and Lee, M.R. (2018). Current Status of the Use of Antibiotics and the Antimicrobial Resistance in the Chilean Salmon Farms. *Frontiers in Microbiology* 9(1284). doi: 10.3389/fmicb.2018.01284.

Nhu Thuy, T., Schaubroeck, T., De Meester, S., Duyvejonck, M., Sorgeloos, P., and Dewulf, J. (2015). Resource consumption assessment of Pangasius fillet products from Vietnamese aquaculture to European retailers. *Journal of Cleaner Production* (0). doi: <http://dx.doi.org/10.1016/j.jclepro.2015.03.030>.

Parker, R. (2017). Implications of high animal by-product feed inputs in life cycle assessments of farmed Atlantic salmon. *The International Journal of Life Cycle Assessment***,** 1-13.

Parker, R.W., Blanchard, J.L., Gardner, C., Green, B.S., Hartmann, K., Tyedmers, P.H., et al. (2018). Fuel use and greenhouse gas emissions of world fisheries. *Nature Climate Change* 8(4)**,** 333.

Parker, R.W.R., Hartmann, K., Green, B.S., Gardner, C., and Watson, R.A. (2015). Environmental and economic dimensions of fuel use in Australian fisheries. *Journal of Cleaner Production* 87(0)**,** 78-86. doi: <http://dx.doi.org/10.1016/j.jclepro.2014.09.081>.

Patterson, H., Noriega, R., Georgeson, L., Larcombe, J., and Curtotti, R. (2017). "Fishery status reports 2017". Australian Bureau of Agricultural and Resource Economics and Sciences, Canberra. CC BY 4.0).

Peet, C. (2014). "Monterey Bay Aquarium Seafood Watch: Barramundi (Lates calcifer)". Monterey Bay Aquarium ).

Pelletier, N., and Tyedmers, P. (2010). Life Cycle Assessment of Frozen Tilapia Fillets From Indonesian Lake-Based and Pond-Based Intensive Aquaculture Systems. *Journal of Industrial Ecology* 14(3)**,** 467-481. doi: 10.1111/j.1530-9290.2010.00244.x.

Pernet, F., Lupo, C., Bacher, C., and Whittington, R.J. (2016). Infectious diseases in oyster aquaculture require a new integrated approach. *Phil. Trans. R. Soc. B* 371(1689)**,** 20150213.

Pons, M., Branch, T.A., Melnychuk, M.C., Jensen, O.P., Brodziak, J., Fromentin, J.M., et al. (2017). Effects of biological, economic and management factors on tuna and billfish stock status. *Fish and Fisheries* 18(1)**,** 1-21.

Poore, J., and Nemecek, T. (2018). Reducing food’s environmental impacts through producers and consumers. *Science* 360(6392)**,** 987-992. doi: 10.1126/science.aaq0216.

Rico, A., Phu, T.M., Satapornvanit, K., Min, J., Shahabuddin, A.M., Henriksson, P.J.G., et al. (2013). Use of veterinary medicines, feed additives and probiotics in four major internationally traded aquaculture species farmed in Asia. *Aquaculture* 412-413**,** 231-243. doi: <https://doi.org/10.1016/j.aquaculture.2013.07.028>.

Rico, A., and Van den Brink, P.J. (2014). Probabilistic risk assessment of veterinary medicines applied to four major aquaculture species produced in Asia. *Science of The Total Environment* 468-469**,** 630-641. doi: <https://doi.org/10.1016/j.scitotenv.2013.08.063>.

Stewardson, C., Andrews, A., Ashby, C., Haddon, M., Hartmann, K., Hone, P., et al. (2016). "Status of Australian fish stocks reports 2016". (Canberra: Fisheries Research and Development Corporation).

Tuck, G.N., Knuckey, I., and Klaer, N. (2013). *Informing the review of the Commonwealth Policy on Fisheries Bycatch through assessing trends in bycatch of key Commonwealth fisheries.* CSIRO.

Vázquez-Rowe, I., Moreira, M., and Feijoo, G. (2012). Environmental assessment of frozen common octopus (Octopus vulgaris) captured by Spanish fishing vessels in the Mauritanian EEZ. *Marine Policy* 36(1)**,** 180-188. doi: <http://dx.doi.org/10.1016/j.marpol.2011.05.002>.

Watts, J., Schreier, H., Lanska, L., and Hale, M. (2017). The Rising Tide of Antimicrobial Resistance in Aquaculture: Sources, Sinks and Solutions. *Marine Drugs* 15(6)**,** 158.

White, A. (2012). "Life Cycle Assessment (LCA) for Tassal, FY2011-12". (Hobart, Australia: Tassal).

White, C.A., Nichols, P.D., Ross, D.J., and Dempster, T. (2017). Dispersal and assimilation of an aquaculture waste subsidy in a low productivity coastal environment. *Marine Pollution Bulletin* 120(1)**,** 309-321. doi: <https://doi.org/10.1016/j.marpolbul.2017.05.042>.

Wilson, A., Magill, S., and Black, K.D. (2009). Review of environmental impact assessment and monitoring in salmon aquaculture. *FAO Fisheries and Aquaculture Technical Paper* 527**,** 455-535.
